# Supplementary material for: A longitudinal psychometric evaluation of a context-sensitive positive health questionnaire for measuring broad health in Dutch adults
Source: J Patient Rep Outcomes. 2026 Jun 2;10:136. doi: 10.1186/s41687-026-01098-x (PMC13454085; doi:10.1186/s41687-026-01098-x)
Supplement: Supplementary file 1 [file 41687_2026_1098_MOESM1_ESM.docx]

**Appendix A**

- **Table A1.** Socio-demographic characteristics of participants by type of dataset
- **Table A2.** Socio-demographic characteristics of participants relative to Dutch population
- **Table A3.** Parallel Analysis (including scree plot)
- **Table A4:** Model fit and variance explained for a series of Exploratory Factor Analyses
- **Table A5.** Standardized weights of the exploratory eight-factor model using oblimin rotated factors
- **Table A6a-l.** Results of the known-group analyses: t-test and ANOVA on CPHQ scores and several characteristics of participants
- **Table A7a-i.** Results of the test-retest analyses: mixed models on CPHQ scores across time
- **Table A8.** Results of the test-retest analyses: weighted Kappa analyses on CPHQ scores
- **Table A9a-b.** Complete item intercorrelation for subsample T1 and T2

| **Table A1.** Socio-demographic characteristics of participants by type of dataset | | | | |
| --- | --- | --- | --- | --- |
|  | **CFA (N=501)** | **EFA (N=500)** | **Total (N=1001)** | **p-value** |
| **Sex** |  |  |  |  |
| Male | 244 (48.7%) | 250 (50.0%) | 494 (49.4%) | 0.919 |
| Female | 257 (51.3%) | 250 (50.0%) | 507 (50.6%) |  |
| **Age** |  |  |  |  |
| 18-29 years | 80 (16.0%) | 72 (14.4%) | 152 (15.2%) | 0.913 |
| 30-44 years | 96 (19.2%) | 104 (20.8%) | 200 (20.0%) |  |
| 45-59 years | 122 (24.4%) | 136 (27.2%) | 258 (25.8%) |  |
| 60 years and above | 203 (40.5%) | 188 (37.6%) | 391 (39.1%) |  |
| **Education level** |  |  |  |  |
| Low | 63 (12.6%) | 48 (9.6%) | 111 (11.1%) | 0.687 |
| Middle | 162 (32.3%) | 165 (33.0%) | 327 (32.7%) |  |
| High | 276 (55.1%) | 287 (57.4%) | 563 (56.2%) |  |
| **Income level** |  |  |  |  |
| Below modal | 115 (23.0%) | 112 (22.4%) | 227 (22.7%) | 0.999 |
| Modal | 69 (13.8%) | 71 (14.2%) | 140 (14.0%) |  |
| Above modal | 313 (62.5%) | 313 (62.6%) | 626 (62.5%) |  |
| Missing | 4 (0.8%) | 4 (0.8%) | 8 (0.8%) |  |
| **Region of residence** |  |  |  |  |
| North | 89 (17.8%) | 96 (19.2%) | 185 (18.5%) | 0.956 |
| Middle | 288 (57.5%) | 275 (55.0%) | 563 (56.2%) |  |
| South | 124 (24.8%) | 129 (25.8%) | 253 (25.3%) |  |
| **SEP** |  |  |  |  |
| Low | 131 (26.1%) | 122 (24.4%) | 253 (25.3%) | 0.974 |
| High | 366 (73.1%) | 373 (74.6%) | 739 (73.8%) |  |
| Unknown | 4 (0.8%) | 5 (1.0%) | 9 (0.9%) |  |
| **Adverse life events** | | | | |
| **Type of event** | | | **Total (N=1001)** | |
| Starting increased caregiving responsibilities | | | 63 (6.3%) | |
| Death of a loved one | | | 169 (16.9%) | |
| Divorce / separation | | | 15 (1.5%) | |
| Moving / relocation | | | 83 (8.3%) | |
| Job loss / dismissal | | | 22 (2.2%) | |
| Accident | | | 23 2.3%) | |
| Mental illness | | | 42 (4.2%) | |
| Physical illness | | | 139 (13.9%) | |
| Other adverse event | | | 38 (3.8%) | |
| **Positive life events** | | | | |
| **Type of event** | | | **Total (N=1001)** | |
| Marriage | | | 48 (4.8%) | |
| Birth of a child | | | 41 (4.1%) | |
| Stopping reduced caregiving responsibilities | | | 26 (2.6%) | |
| Moving in together / Cohabitation | | | 31 (3.1%) | |
| New job / career opportunity | | | 142 (14.2%) | |
| Other positive event | | | 321 (2.1%) | |
| *Note.* SEP = socioeconomic position. | | | | |

| **Table A2.** Socio-demographic characteristics of participants relative to Dutch population | | | | |
| --- | --- | --- | --- | --- |
|  | **T0** | **T1** | **T2** | **SN 2023*** |
|  | **N=1001** | **N=714** | **N=543** |  |
| **Sex** |  |  |  |  |
| Male | 49% | 51% | 51% | 49% |
| Female | 51% | 49% | 49% | 51% |
| **Age** |  |  |  |  |
| 18-29 years | 15% | 17% | 18% | 19% |
| 30-44 years | 20% | 22% | 21% | 23% |
| 45-59 years | 26% | 27% | 27% | 26% |
| 60 years and above | 39% | 34% | 34% | 32% |
| **Region of residence** |  |  |  |  |
| North | 19% | 19% | 19% | 19% |
| Middle | 56% | 56% | 57% | 58% |
| South | 25% | 25% | 25% | 24% |
| **SEP** |  |  |  |  |
| Low | 25% | 28% | 28% | 27% |
| High | 74% | 72% | 72% | 73% |
| Unknown | 1% | - |  | - |
| *Note.* *Numbers based on SN (Statistics Netherlands); SEP = socioeconomic status. | | | | |

| **Table A3.** Parallel analyses | | |
| --- | --- | --- |
| **Factors** | **Actual** | **Simulated** |
| 1 | 9.881641 | 0.633359 |
| 2 | 1.396466 | 0.422698 |
| 3 | 1.242953 | 0.368379 |
| 4 | 0.60676 | 0.323449 |
| 5 | 0.412056 | 0.285269 |
| 6 | 0.39626 | 0.248348 |
| 7 | 0.273896 | 0.215037 |
| 8 | 0.194532 | 0.182776 |
| 9 | 0.092237 | 0.151257 |
| 10 | 0.006139 | 0.122537 |
| 11 | -0.02995 | 0.093488 |
| 12 | -0.07773 | 0.065539 |
| 13 | -0.10872 | 0.038964 |
| 14 | -0.13986 | 0.012855 |
| 15 | -0.16474 | -0.01268 |

| **Table A4**: Model fit and variance explained for a series of Exploratory Factor Analyses | | | | | |
| --- | --- | --- | --- | --- | --- |
| **Number of factors** | **CFI** | **TLI** | **RMSEA** | **RMSR** | **Cumulative variance explained (%)** |
| 1 | 0.66 | 0.63 | 0.12 | 0.09 | 35.30 |
| 2 | 0.76 | 0.72 | 0.10 | 0.07 | 41.14 |
| 3 | 0.83 | 0.79 | 0.09 | 0.05 | 46.40 |
| 4 | 0.87 | 0.82 | 0.08 | 0.04 | 49.25 |
| 5 | 0.91 | 0.86 | 0.07 | 0.04 | 52.38 |
| 6 | 0.93 | 0.88 | 0.07 | 0.03 | 54.72 |
| 7 | 0.95 | 0.91 | 0.06 | 0.02 | 56.74 |
| 8 | 0.97 | 0.95 | 0.05 | 0.02 | 59.16 |
| 9 | 0.98 | 0.96 | 0.04 | 0.02 | 61.04 |
| 10* | 0.99 | 0.97 | 0.04 | 0.01 | 61.86 |
| 11 | 0.97 | 0.91 | 0.06 | 0.01 | 62.99 |
| 12 | 0.99 | 0.98 | 0.03 | 0.01 | 64.22 |
| 13* | 0.99 | 0.96 | 0.04 | 0.01 | 65.30 |
| 14* | 1.00 | 0.99 | 0.02 | 0.01 | 67.31 |
| 15* | 1.00 | 0.98 | 0.03 | 0.01 | 69.22 |
| *Note.* CFI = Comparative Fit Index; TLI = Tucker-Lewis Index; RMSEA = Root Mean Square Error of Approximation; SRMR = Standardized Root Mean Square Residual | | | | | |

| **Table A5.** Standardized weights of the exploratory eight-factor model using oblimin rotated factors | | | | | | | | | | |
| --- | --- | --- | --- | --- | --- | --- | --- | --- | --- | --- |
|  | **F1** | **F2** | **F3** | **F4** | **F5** | **F6** | **F7** | **F8** | **h2** | **com** |
| I feel calm | **0.780** | 0.010 | -0.030 | 0.150 | 0.030 | -0.010 | 0.000 | -0.070 | 0.71 | 1.1 |
| I can relax | **0.730** | 0.030 | 0.060 | -0.060 | 0.040 | 0.050 | 0.050 | 0.090 | 0.65 | 1.1 |
| I can do what I think is important | 0.360 | 0.060 | 0.180 | -0.010 | 0.090 | 0.280 | -0.010 | 0.190 | 0.56 | 3.2 |
| I can express my boundaries | 0.350 | 0.180 | -0.080 | 0.120 | -0.020 | -0.120 | 0.330 | -0.150 | 0.36 | 3.5 |
| I can cope with setbacks | 0.340 | 0.100 | 0.100 | 0.060 | -0.050 | -0.030 | 0.320 | -0.080 | 0.38 | 2.6 |
| I feel accepted | 0.020 | **0.730** | 0.090 | -0.050 | -0.040 | 0.120 | 0.050 | 0.070 | 0.71 | 1.1 |
| I feel that I belong | 0.000 | **0.670** | 0.030 | 0.070 | 0.250 | -0.040 | 0.010 | -0.020 | 0.74 | 1.3 |
| I feel excluded* | 0.070 | **0.580** | 0.070 | 0.060 | 0.100 | 0.040 | -0.030 | -0.050 | 0.54 | 1.2 |
| I feel happy | 0.170 | 0.080 | **0.600** | 0.120 | 0.080 | 0.050 | -0.030 | -0.020 | 0.72 | 1.3 |
| I feel my life is meaningful | -0.040 | 0.170 | **0.590** | 0.160 | 0.060 | -0.020 | 0.100 | -0.070 | 0.65 | 1.4 |
| I feel grateful for my life | -0.070 | 0.210 | **0.510** | 0.080 | 0.060 | 0.030 | 0.070 | 0.000 | 0.50 | 1.5 |
| I can enjoy | 0.360 | 0.050 | **0.450** | -0.160 | 0.050 | 0.040 | 0.060 | 0.250 | 0.65 | 3.0 |
| I have goals and ideals I want to achieve | -0.090 | -0.240 | 0.280 | 0.210 | 0.240 | 0.110 | 0.260 | -0.100 | 0.37 | 5.7 |
| I feel fit | 0.060 | 0.040 | 0.010 | **0.800** | -0.020 | 0.070 | -0.020 | 0.120 | 0.81 | 1.1 |
| I feel healthy | 0.050 | -0.010 | 0.100 | **0.760** | 0.000 | 0.060 | 0.040 | 0.050 | 0.77 | 1.1 |
| I feel good about myself | 0.380 | 0.040 | 0.270 | 0.390 | 0.090 | 0.010 | -0.050 | -0.050 | 0.74 | 3.0 |
| I have people who support me | 0.010 | 0.000 | -0.010 | -0.030 | **0.900** | 0.020 | 0.020 | 0.000 | 0.83 | 1.0 |
| I feel that people support me | 0.030 | 0.300 | -0.030 | -0.020 | **0.560** | 0.050 | 0.100 | -0.020 | 0.69 | 1.7 |
| I have people with I can do fun things with | 0.040 | 0.060 | 0.120 | 0.000 | **0.550** | 0.060 | -0.100 | 0.140 | 0.49 | 1.4 |
| I have money to do the things that I find important | 0.000 | 0.000 | -0.020 | 0.000 | 0.000 | **1.020** | -0.030 | -0.030 | 0.99 | 1.0 |
| I have money to live a healthy life | 0.000 | 0.030 | -0.010 | 0.050 | 0.010 | **0.760** | 0.070 | 0.010 | 0.66 | 1.0 |
| I feel safe where I live | -0.060 | 0.220 | 0.130 | -0.030 | -0.090 | 0.280 | 0.190 | 0.090 | 0.29 | 3.8 |
| I can understand information about my health | 0.040 | 0.060 | -0.040 | -0.120 | 0.000 | 0.120 | **0.570** | 0.080 | 0.41 | 1.3 |
| I can find help for my health | 0.020 | -0.040 | 0.080 | 0.010 | 0.150 | 0.090 | **0.550** | 0.090 | 0.52 | 1.3 |
| I can move | 0.010 | 0.080 | -0.090 | 0.330 | 0.050 | 0.030 | 0.050 | **0.610** | 0.67 | 1.7 |
| I can be active in my environment | -0.010 | -0.030 | 0.100 | 0.220 | 0.110 | 0.030 | 0.260 | **0.430** | 0.58 | 2.6 |
| I can work/volunteer or provide informal care | -0.080 | 0.010 | 0.080 | 0.230 | 0.090 | 0.000 | 0.120 | 0.320 | 0.30 | 2.6 |
| I have confidence in society | 0.050 | 0.190 | 0.120 | 0.140 | 0.040 | 0.110 | 0.190 | -0.260 | 0.30 | 4.6 |
| *Note.* * Negative formulation; F1 = Mental Relaxation; F2 = Social Acceptance; F3 = Wellbeing; F4 = Vitality; F5 = Social Support; F6 = Financial Resources; F7 = Health Literacy; F8 = Mobility. | | | | | | | | | | |

| **Table A6a.** Results of known-groups analyses: independent samples t-test on CPHQ scores and gender | | | | | | |
| --- | --- | --- | --- | --- | --- | --- |
|  |  | **Male (N=494)** | **Female (N=507)** | **Total (N=1001)** | **p-value** | **Cohen’s D**  **[95% CI]** |
| **CPHQ mental relaxation** |  |  |  |  | <0.001 | 0.29 [0.17-0.42] |
| Mean (SD) |  | 73.1 (16.3) | 68.2 (16.7) | 70.6 (16.7) |  |  |
| **CPHQ social acceptance** |  |  |  |  | <0.001 | 0.25 [0.13-0.38] |
| Mean (SD) |  | 78.3 (13.5) | 74.5 (16.2) | 76.4 (15.0) |  |  |
| **CPHQ wellbeing** |  |  |  |  | 0.146 | 0.09 [-0.03-0.22] |
| Mean (SD) |  | 75.5 (14.8) | 74.1 (15.3) | 74.8 (15.0) |  |  |
| **CPHQ vitality** |  |  |  |  | 0.092 | 0.11 [-0.02-0.23] |
| Mean (SD) |  | 67.8 (19.1) | 65.8 (20.0) | 66.8 (19.6) |  |  |
| **CPHQ social support** |  |  |  |  | 0.107 | -0.10 [-0.23-0.02] |
| Mean (SD) |  | 75.4 (13.3) | 76.8 (14.8) | 76.1 (14.1) |  |  |
| **CPHQ financial resources** |  |  |  |  | 0.483 | 0.04 [-0.08-0.13] |
| Mean (SD) |  | 80.3 (16.4) | 79.6 (17.6) | 80.0 (17.0) |  |  |
| **CPHQ health literacy** |  |  |  |  | 0.909 | 0.01 [-0.12-0.13] |
| Mean (SD) |  | 81.4 (11.8) | 81.3 (13.0) | 81.3 (12.4) |  |  |
| **CPHQ mobility** |  |  |  |  | 0.710 | -0.02 [-0.15-0.10] |
| Mean (SD) |  | 77.0 (14.3) | 77.3 (14.9) | 77.2 (14.6) |  |  |
| **CPHQ total score** |  |  |  |  | 0.045 | 0.13 [0.00-0.25] |
| Mean (SD) |  | 76.1 (10.5) | 74.7 (11.5) | 75.4 (11.0) |  |  |

| **Table A6b.** Results of known-groups analyses: independent samples t-test on CPHQ scores and age | | | | | |
| --- | --- | --- | --- | --- | --- |
|  | **18-64 (N=728)** | **65 and above (N=273)** | **Total (N=1001)** | **p-value** | **Cohen’s D**  **[95% CI]** |
| **CPHQ mental relaxation** |  |  |  | <0.001 | -0.27 [-0.41--0.13] |
| Mean (SD) | 69.4 (17.4) | 73.9 (14.0) | 70.6 (16.7) |  |  |
| **CPHQ social acceptance** |  |  |  | 0.014 | -0.16 [-0.3--0.02] |
| Mean (SD) | 75.7 (15.7) | 78.1 (13.1) | 76.4 (15.0) |  |  |
| **CPHQ wellbeing** |  |  |  | 0.714 | -0.02 [-0.16-0.11] |
| Mean (SD) | 74.7 (15.6) | 75.0 (13.3) | 74.8 (15.0) |  |  |
| **CPHQ vitality** |  |  |  | 0.033 | 0.15 [0.01-0.29] |
| Mean (SD) | 67.6 (19.7) | 64.7 (19.1) | 66.8 (19.6) |  |  |
| **CPHQ social support** |  |  |  | <0.001 | 0.23 [0.10-0.37] |
| Mean (SD) | 77.0 (14.3) | 73.7 (13.3) | 76.1 (14.1) |  |  |
| **CPHQ financial resources** |  |  |  | 0.016 | 0.17 [0.03-0.31] |
| Mean (SD) | 80.8 (16.9) | 77.8 (17.1) | 80.0 (17.0) |  |  |
| **CPHQ health literacy** |  |  |  | 0.003 | 0.20 [0.06-0.34] |
| Mean (SD) | 82.0 (12.9) | 79.5 (11.0) | 81.3 (12.4) |  |  |
| **CPHQ mobility** |  |  |  | <0.001 | 0.34 [0.20-0.48] |
| Mean (SD) | 78.5 (14.2) | 73.6 (15.1) | 77.2 (14.6) |  |  |
| **CPHQ total score** |  |  |  | 0.125 | 0.10 [-0.04-0.24] |
| Mean (SD) | 75.7 (11.3) | 74.6 (10.2) | 75.4 (11.0) |  |  |

| **Table A6c.** Results of known-groups analyses independent samples t-test on CPHQ scores and migration background | | | | | |
| --- | --- | --- | --- | --- | --- |
|  | **Both parents born in the Netherlands (N=962)** | **Both parents born outside the Netherlands (N=36)** | **Total (N=998)** | **p-value** | **Cohen’s D**  **[95% CI]** |
| **CPHQ mental relaxation** |  |  |  | 0.069 | 0.22 [0.01-0.44] |
| Mean (SD) | 71.0 (16.5) | 67.3 (18.4) | 70.7 (16.7) |  |  |
| **CPHQ social acceptance** |  |  |  | 0.069 | 0.22 [0.00-0.44] |
| Mean (SD) | 76.7 (14.8) | 73.4 (16.3) | 76.4 (15.0) |  |  |
| **CPHQ wellbeing** |  |  |  | 0.153 | 0.17 [-0.04-0.39] |
| Mean (SD) | 75.1 (14.8) | 72.5 (16.4) | 74.8 (15.0) |  |  |
| **CPHQ vitality** |  |  |  | 0.112 | 0.18 [-0.04-0.40] |
| Mean (SD) | 67.2 (19.5) | 63.6 (19.9) | 66.8 (19.5) |  |  |
| **CPHQ social support** |  |  |  | 0.104 | 0.17 [-0.05-0.39] |
| Mean (SD) | 76.4 (14.2) | 74.0 (13.0) | 76.2 (14.1) |  |  |
| **CPHQ financial resources** |  |  |  | 0.010 | 0.30 [0.08-0.51] |
| Mean (SD) | 80.4 (16.9) | 75.4 (17.1) | 80.0 (17.0) |  |  |
| **CPHQ health literacy** |  |  |  | 0.011 | 0.33 [0.11-0.55] |
| Mean (SD) | 81.7 (12.2) | 77.7 (14.3) | 81.4 (12.4) |  |  |
| **CPHQ mobility** |  |  |  | 0.040 | 0.23 [0.01-0.45] |
| Mean (SD) | 77.5 (14.6) | 74.2 (14.6) | 77.2 (14.6) |  |  |
| **CPHQ total score** |  |  |  | 0.007 | 0.32 [0.10-0.54] |
| Mean (SD) | 75.7 (10.9) | 72.2 (11.5) | 75.4 (11.0) |  |  |

| **Table A6d.** Results of known-groups analyses: ANOVA on CPHQ scores and educational level | | | | | | |
| --- | --- | --- | --- | --- | --- | --- |
|  | **Low (N=111)** | **Middle (N=327)** | **High (N=563)** | **Total (N=1001)** | **p-value** | **Eta2 [95% CI]** |
| **CPHQ mental relaxation** |  |  |  |  | 0.173 | 0.00 [0.00-1.00] |
| Mean (SD) | 68.1 (16.2) | 71.6 (17.1) | 70.6 (16.5) | 70.6 (16.7) |  |  |
| **CPHQ social acceptance** |  |  |  |  | <0.001 | 0.01 [0.00-1.00] |
| Mean (SD) | 71.5 (15.5) | 76.1 (15.3) | 77.4 (14.6) | 76.4 (15.0) |  |  |
| **CPHQ wellbeing** |  |  |  |  | <0.001 | 0.02 [0.01-1.00] |
| Mean (SD) | 69.3 (16.0) | 74.7 (16.1) | 75.9 (13.9) | 74.8 (15.0) |  |  |
| **CPHQ vitality** |  |  |  |  | <0.001 | 0.05 [0.03-1.00] |
| Mean (SD) | 55.5 (20.1) | 65.1 (19.5) | 70.0 (18.6) | 66.8 (19.6) |  |  |
| **CPHQ social support** |  |  |  |  | <0.001 | 0.05 [0.03-1.00] |
| Mean (SD) | 67.9 (15.2) | 75.4 (14.1) | 78.2 (13.3) | 76.1 (14.1) |  |  |
| **CPHQ financial resources** |  |  |  |  | <0.001 | 0.15 [0.12-1.00] |
| Mean (SD) | 64.8 (18.2) | 76.5 (17.5) | 85.0 (13.9) | 80.0 (17.0) |  |  |
| **CPHQ health literacy** |  |  |  |  | <0.001 | 0.08 [0.05-1.00] |
| Mean (SD) | 73.9 (10.0) | 79.1 (12.2) | 84.1 (12.1) | 81.3 (12.4) |  |  |
| **CPHQ mobility** |  |  |  |  | <0.001 | 0.06 [0.04-1.00] |
| Mean (SD) | 68.1 (14.4) | 76.1 (14.8) | 79.6 (13.7) | 77.2 (14.6) |  |  |
| **CPHQ total score** |  |  |  |  | <0.001 | 0.08 [0.06-1.00] |
| Mean (SD) | 67.4 (10.7) | 74.3 (11.3) | 77.6 (10.1) | 75.4 (11.0) |  |  |

| **Table A6e.** Results of known-groups analyses: ANOVA on CPHQ scores and income | | | | | | |
| --- | --- | --- | --- | --- | --- | --- |
|  | **Below modal (N=227)** | **Modal (N=140)** | **Above modal (N=626)** | **Total (N=993)** | **p-value** | **Eta2 [95% CI]** |
| **CPHQ mental relaxation** |  |  |  |  | 0.097 | 0.00 [0.00-1.00] |
| Mean (SD) | 68.6 (16.7) | 71.4 (16.4) | 71.2 (16.8) | 70.7 (16.7) |  |  |
| **CPHQ social acceptance** |  |  |  |  | <0.001 | 0.03 [0.02-1.00] |
| Mean (SD) | 71.4 (15.9) | 76.5 (13.3) | 78.0 (14.8) | 76.3 (15.1) |  |  |
| **CPHQ wellbeing** |  |  |  |  | <0.001 | 0.03 [0.02-1.00] |
| Mean (SD) | 69.9 (17.0) | 76.5 (13.6) | 76.2 (14.3) | 74.8 (15.1) |  |  |
| **CPHQ vitality** |  |  |  |  | <0.001 | 0.06 [0.04-1.00] |
| Mean (SD) | 58.3 (21.4) | 67.9 (19.2) | 69.7 (17.9) | 66.9 (19.5) |  |  |
| **CPHQ social support** |  |  |  |  | <0.001 | 0.05 [0.03-1.00] |
| Mean (SD) | 70.5 (15.7) | 75.2 (12.1) | 78.4 (13.4) | 76.1 (14.1) |  |  |
| **CPHQ financial resources** |  |  |  |  | <0.001 | 0.20 [0.16-1.00] |
| Mean (SD) | 66.5 (19.2) | 79.2 (15.0) | 85.0 (13.6) | 79.9 (17.0) |  |  |
| **CPHQ health literacy** |  |  |  |  | <0.001 | 0.05 [0.03-1.00] |
| Mean (SD) | 76.5 (11.9) | 81.3 (13.4) | 83.1 (12.0) | 81.3 (12.5) |  |  |
| **CPHQ mobility** |  |  |  |  | <0.001 | 0.06 [0.04-1.00] |
| Mean (SD) | 70.5 (16.0) | 78.3 (14.4) | 79.4 (13.3) | 77.2 (14.6) |  |  |
| **CPHQ total score** |  |  |  |  | <0.001 | 0.10 [0.07-1.00] |
| Mean (SD) | 69.0 (12.0) | 75.8 (10.3) | 77.6 (9.93) | 75.4 (11.1) |  |  |

| **Table A6f.** Results of known-groups analyses: independent samples t-test on CPHQ scores and socioeconomic status | | | | | |
| --- | --- | --- | --- | --- | --- |
|  | **Low (N=253)** | **High (N=739)** | **Total (N=992)** | **p-value** | **Cohen’s D**  **[95% CI]** |
| **CPHQ mental relaxation** |  |  |  | 0.056 | -0.15 [-0.28-0.00] |
| Mean (SD) | 68.9 (16.8) | 71.3 (16.7) | 70.7 (16.7) |  |  |
| **CPHQ social acceptance** |  |  |  | <0.001 | -0.35 [-0.50--0.21] |
| Mean (SD) | 72.4 (15.7) | 77.7 (14.6) | 76.3 (15.1) |  |  |
| **CPHQ wellbeing** |  |  |  | <0.001 | -0.37 [-0.5--0.23] |
| Mean (SD) | 70.7 (16.8) | 76.2 (14.2) | 74.8 (15.1) |  |  |
| **CPHQ vitality** |  |  |  | <0.001 | -0.61 [-0.75--0.46] |
| Mean (SD) | 58.3 (21.0) | 69.8 (18.1) | 66.9 (19.5) |  |  |
| **CPHQ social support** |  |  |  | <0.001 | -0.50 [-0.65--0.36] |
| Mean (SD) | 70.9 (15.3) | 77.9 (13.3) | 76.1 (14.1) |  |  |
| **CPHQ financial resources** |  |  |  | <0.001 | -1.11 [-1.26--0.95] |
| Mean (SD) | 67.3 (18.8) | 84.3 (14.0) | 79.9 (17.0) |  |  |
| **CPHQ health literacy** |  |  |  | <0.001 | -0.56 [-0.71--0.42] |
| Mean (SD) | 76.3 (11.7) | 83.1 (12.3) | 81.4 (12.5) |  |  |
| **CPHQ mobility** |  |  |  | <0.001 | -0.60 [-0.75--0.46] |
| Mean (SD) | 70.8 (15.8) | 79.4 (13.5) | 77.2 (14.6) |  |  |
| **CPHQ total score** |  |  |  | <0.001 | -0.76 [-0.91--0.61] |
| Mean (SD) | 69.5 (11.8) | 77.4 (10.0) | 75.4 (11.1) |  |  |

| **Table A6g.** Results of known-groups analyses: independent samples t-test on CPHQ scores and household composition | | | | | |
| --- | --- | --- | --- | --- | --- |
|  | **Without partner (N=276)** | **With partner (N=725)** | **Total (N=1001)** | **p-value** | **Cohen’s D**  **[95% CI]** |
| **CPHQ mental relaxation** |  |  |  | 0.160 | -0.10 [-0.24-0.03] |
| Mean (SD) | 69.4 (17.9) | 71.1 (16.2) | 70.6 (16.7) |  |  |
| **CPHQ social acceptance** |  |  |  | <0.001 | -0.36 [-0.50--0.22] |
| Mean (SD) | 72.5 (16.1) | 77.8 (14.4) | 76.4 (15.0) |  |  |
| **CPHQ wellbeing** |  |  |  | <0.001 | -0.36 [-0.50--0.22] |
| Mean (SD) | 70.9 (16.4) | 76.3 (14.2) | 74.8 (15.0) |  |  |
| **CPHQ vitality** |  |  |  | 0.0614 | -0.14 [-0.28-0.00] |
| Mean (SD) | 64.8 (21.9) | 67.6 (18.6) | 66.8 (19.6) |  |  |
| **CPHQ social support** |  |  |  | <0.001 | -0.27 [-0.41--0.13] |
| Mean (SD) | 73.4 (15.8) | 77.2 (13.3) | 76.1 (14.1) |  |  |
| **CPHQ financial resources** |  |  |  | <0.001 | -0.33 [-0.47--0.79] |
| Mean (SD) | 76.0 (19.7) | 81.5 (15.6) | 80.0 (17.0) |  |  |
| **CPHQ health literacy** |  |  |  | 0.172 | -0.10 [-0.24-0.04] |
| Mean (SD) | 80.4 (13.0) | 81.7 (12.2) | 81.3 (12.4) |  |  |
| **CPHQ mobility** |  |  |  | 0.152 | -0.11 [-0.25-0.03] |
| Mean (SD) | 76.0 (15.9) | 77.6 (14.1) | 77.2 (14.6) |  |  |
| **CPHQ total score** |  |  |  | <0.001 | -0.31 [-0.45--0.17] |
| Mean (SD) | 72.9 (12.4) | 76.3 (10.3) | 75.4 (11.0) |  |  |

| **Table A6h.** Results of known-groups analyses: independent samples t-test on CPHQ scores and adverse life events | | | | | |
| --- | --- | --- | --- | --- | --- |
|  | **No (N=571)** | **Yes (N=430)** | **Total (N=1001)** | **p-value** | **Cohen’s D**  **[95% CI]** |
| **CPHQ mental relaxation** |  |  |  | <0.001 | 0.37 [0.24-0.50] |
| Mean (SD) | 73.2 (15.3) | 67.2 (17.8) | 70.6 (16.7) |  |  |
| **CPHQ social acceptance** |  |  |  | 0.047 | 0.13 [0.00-0.25] |
| Mean (SD) | 77.2 (14.4) | 75.3 (15.8) | 76.4 (15.0) |  |  |
| **CPHQ wellbeing** |  |  |  | 0.007 | 0.18 [0.05-0.30] |
| Mean (SD) | 75.9 (14.5) | 73.3 (15.5) | 74.8 (15.0) |  |  |
| **CPHQ vitality** |  |  |  | <0.001 | 0.35 [0.23-0.48] |
| Mean (SD) | 69.7 (14.5) | 62.9 (20.5) | 66.8 (19.6) |  |  |
| **CPHQ social support** |  |  |  | 0.897 | -0.01 [-0.13-0.12] |
| Mean (SD) | 76.1 (14.0) | 76.2 (14.3) | 76.1 (14.1) |  |  |
| **CPHQ financial resources** |  |  |  | 0.692 | 0.03 [-0.10-0.15] |
| Mean (SD) | 80.1 (16.5) | 79.7 (17.7) | 80.0 (17.0) |  |  |
| **CPHQ health literacy** |  |  |  | 0.620 | 0.03 [-0.09-0.16] |
| Mean (SD) | 81.5 (12.1) | 81.1 (12.9) | 81.3 (12.4) |  |  |
| **CPHQ mobility** |  |  |  | 0.004 | 0.19 [0.06-0.31] |
| Mean (SD) | 78.3 (13.8) | 75.6 (15.5) | 77.2 (14.6) |  |  |
| **CPHQ total score** |  |  |  | <0.001 | 0.24 [0.11-0.36] |
| Mean (SD) | 76.5 (10.4) | 73.9 (11.7) | 75.4 (11.0) |  |  |

| **Table A6i.** Results of known-groups analyses: ANOVA on CPHQ scores and number of adverse life events | | | | | | |
| --- | --- | --- | --- | --- | --- | --- |
|  | **No life events (N=571)** | **One life event (N=300)** | **2 or more life events (N=130)** | **Total (N=1001)** | **p-value** | **Eta2 [95% CI]** |
| **CPHQ mental relaxation** |  |  |  |  | <0.001 | 0.04 [0.02-1.00] |
| Mean (SD) | 73.2 (15.3) | 68.4 (17.2) | 64.3 (18.7) | 70.6 (16.7) |  |  |
| **CPHQ social acceptance** |  |  |  |  | 0.026 | 0.01 [0.00-1.00] |
| Mean (SD) | 77.2 (14.4) | 76.1 (15.1) | 73.3 (17.3) | 76.4 (15.0) |  |  |
| **CPHQ wellbeing** |  |  |  |  | <0.001 | 0.01 [0.00-1.00] |
| Mean (SD) | 75.9 (14.5) | 74.5 (14.8) | 70.4 (16.8) | 74.8 (15.0) |  |  |
| **CPHQ vitality** |  |  |  |  | <0.001 | 0.03 [0.02-1.00] |
| Mean (SD) | 69.7 (18.3) | 64.2 (20.6) | 59.8 (20.0) | 66.8 (19.6) |  |  |
| **CPHQ social support** |  |  |  |  | 0.590 | 0.00 [0.00-1.00] |
| Mean (SD) | 76.1 (14.0) | 76.6 (13.6) | 75.1 (15.8) | 76.1 (14.1) |  |  |
| **CPHQ financial resources** |  |  |  |  | 0.726 | 0.01 [0.00-1.00] |
| Mean (SD) | 80.1 (16.5) | 80.1 (17.2) | 78.8 (18.9) | 80.0 (17.0) |  |  |
| **CPHQ health literacy** |  |  |  |  | 0.423 | 0.00 [0.00-1.00] |
| Mean (SD) | 81.5 (12.1) | 81.6 (12.2) | 80.0 (14.5) | 81.3 (12.4) |  |  |
| **CPHQ mobility** |  |  |  |  | 0.003 | 0.01 [0.00-1.00] |
| Mean (SD) | 78.3 (13.8) | 76.4 (14.9) | 73.8 (16.7) | 77.2 (14.6) |  |  |
| **CPHQ total score** |  |  |  |  | <0.001 | 0.02 [0.01-1.00] |
| Mean (SD) | 76.5 (10.4) | 74.7 (11.2) | 72.0 (12.6) | 75.4 (11.0) |  |  |

| **Table A6j.** Results of known-groups analyses: independent samples t-test on CPHQ scores and positive life events | | | | | |
| --- | --- | --- | --- | --- | --- |
|  | **No (N=754)** | **Yes (N=247)** | **Total (N=1001)** | **p-value** | **Cohen’s D**  **[95% CI]** |
| **CPHQ mental relaxation** |  |  |  | 0.121 | 0.11 [-0.03-0.26] |
| Mean (SD) | 71.1 (16.8) | 69.2 (16.3) | 70.6 (16.7) |  |  |
| **CPHQ social acceptance** |  |  |  | 0.433 | 0.06 [-0.09-0.20] |
| Mean (SD) | 76.6 (15.1) | 75.7 (14.9) | 76.4 (15.0) |  |  |
| **CPHQ wellbeing** |  |  |  | 0.289 | -0.08 [-0.22-0.06] |
| Mean (SD) | 74.5 (14.8) | 75.7 (15.6) | 74.8 (15.0) |  |  |
| **CPHQ vitality** |  |  |  | 0.078 | -0.13 [-0.27-0.02] |
| Mean (SD) | 66.2 (19.6) | 68.7 (19.3) | 66.8 (19.6) |  |  |
| **CPHQ social support** |  |  |  | 0.017 | -0.18 [-0.32--0.03] |
| Mean (SD) | 75.5 (14.0) | 78.0 (14.4) | 76.1 (14.1) |  |  |
| **CPHQ financial resources** |  |  |  | 0.288 | -0.07 [-0.21-0.07] |
| Mean (SD) | 79.7 (17.7) | 80.9 (14.8) | 80.0 (17.0) |  |  |
| **CPHQ health literacy** |  |  |  | 0.044 | -0.15 [-0.29-0.00] |
| Mean (SD) | 80.9 (12.5) | 82.7 (12.1) | 81.3 (12.4) |  |  |
| **CPHQ mobility** |  |  |  | 0.038 | -0.15 [-0.29-0.00] |
| Mean (SD) | 76.6 (14.8) | 78.8 (13.9) | 77.2 (14.6) |  |  |
| **CPHQ total score** |  |  |  | 0.174 | -0.12 [-0.24-0.05] |
| Mean (SD) | 75.1 (11.1) | 76.2 (10.7) | 75.4 (11.0) |  |  |

| **Table A6k.** Results of known-groups analyses: ANVOA on CPHQ scores and number of positive life events | | | | | | |
| --- | --- | --- | --- | --- | --- | --- |
|  | **No life events (N=754)** | **One life event (N=91)** | **2 or more life events (N=156)** | **Total (N=1001)** | **p-value** | **Eta2 [95% CI]** |
| **CPHQ mental relaxation** |  |  |  |  | 0.244 | 0.00 {0.00-1.00} |
| Mean (SD) | 71.1 (16.8) | 70.2 (16.8) | 68.7 (16.0) | 70.6 (16.7) |  |  |
| **CPHQ social acceptance** |  |  |  |  | 0.557 | 0.00 {0.00-1.00} |
| Mean (SD) | 76.6 (15.1) | 76.6 (12.6) | 75.2 (16.1) | 76.4 (15.0) |  |  |
| **CPHQ wellbeing** |  |  |  |  | 0.546 | 0.00 {0.00-1.00} |
| Mean (SD) | 74.5 (14.8) | 75.9 (15.3) | 75.6 (15.8) | 74.8 (15.0) |  |  |
| **CPHQ vitality** |  |  |  |  | 0.131 | 0.00 {0.00-1.00} |
| Mean (SD) | 66.2 (19.6) | 67.0 (19.2) | 69.6 (19.4) | 66.8 (19.6) |  |  |
| **CPHQ social support** |  |  |  |  | 0.040 | 0.01 {0.00-1.00} |
| Mean (SD) | 75.5 (14.0) | 77.1 (14.4) | 78.5 (14.5) | 76.1 (14.1) |  |  |
| **CPHQ financial resources** |  |  |  |  | 0.619 | 0.01 {0.00-1.00} |
| Mean (SD) | 79.7 (17.7) | 81.0 (15.5) | 80.8 (14.4) | 80.0 (17.0) |  |  |
| **CPHQ health literacy** |  |  |  |  | 0.019 | 0.01 {0.00-1.00} |
| Mean (SD) | 80.9 (12.5) | 80.6 (11.7) | 83.9 (12.2) | 81.3 (12.4) |  |  |
| **CPHQ mobility** |  |  |  |  | 0.100 | 0.01 {0.00-1.00} |
| Mean (SD) | 76.6 (14.8) | 77.9 (15.9) | 79.3 (12.5) | 77.2 (14.6) |  |  |
| **CPHQ total score** |  |  |  |  | 0.372 | 0.00 {0.00-1.00} |
| Mean (SD) | 75.1 (11.1) | 75.8 (11.1) | 76.4 (10.6) | 75.4 (11.0) |  |  |

| **Table A6l.** Results of known-groups analyses: independent samples t-test on CPHQ scores and the presence of non-communicable diseases | | | | | |
| --- | --- | --- | --- | --- | --- |
|  | **No (N=482)** | **Yes (N=519)** | **Total (N=1001)** | **p-value** | **Cohen’s D**  **[95% CI]** |
| **CPHQ mental relaxation** |  |  |  | <0.001 | 0.24 [0.11-0.36] |
| Mean (SD) | 2.09 (0.639) | 2.25 (0.685) | 2.17 (0.667) |  |  |
| **CPHQ social acceptance** |  |  |  | <0.001 | 0.22 [0.10-0.35] |
| Mean (SD) | 1.88 (0.585) | 2.01 (0.610) | 1.95 (0.602) |  |  |
| **CPHQ wellbeing** |  |  |  | <0.001 | 0.31 [0.19-0.44] |
| Mean (SD) | 1.91 (0.553) | 2.10 (0.629) | 2.01 (0.601) |  |  |
| **CPHQ vitality** |  |  |  | <0.001 | 0.81 [0.68-0.94] |
| Mean (SD) | 2.02 (0.574) | 2.61 (0.842) | 2.33 (0.783) |  |  |
| **CPHQ social support** |  |  |  | 0.00177 | 0.20 [0.07-0.32] |
| Mean (SD) | 1.90 (0.551) | 2.01 (0.573) | 1.96 (0.565) |  |  |
| **CPHQ financial resources** |  |  |  | <0.001 | 0.28 [0.15-0.40] |
| Mean (SD) | 1.70 (0.565) | 1.89 (0.762) | 1.80 (0.680) |  |  |
| **CPHQ health literacy** |  |  |  | <0.001 | 0.22 [0.10-0.35] |
| Mean (SD) | 1.69 (0.465) | 1.80 (0.520) | 1.75 (0.497) |  |  |
| **CPHQ mobility** |  |  |  | <0.001 | 0.55 [0.42-0.67] |
| Mean (SD) | 1.75 (0.502) | 2.06 (0.615) | 1.91 (0.584) |  |  |
| **CPHQ total score** |  |  |  | <0.001 | 0.52 [0.40-0.65] |
| Mean (SD) | 1.87 (0.375) | 2.09 (0.471) | 1.98 (0.441) |  |  |

| **Table A7a.** Results of the test-retest analysis: mixed models for CPHQ mental relaxation] | | | | | |
| --- | --- | --- | --- | --- | --- |
| **Effect** | **Estimate** | **se** | **t** | **p-value** | **95% CI** |
| **Intercept** | 74.444 | 1.061 | 70.141 | < .001 | [72.359 – 76.528] |
| **Time [ref = T0]** |  |  |  |  |  |
| T1 | -0.184 | 0.515 | -0.357 | 0.721 | [-1.197 – 0.828] |
| T2 | -0.391 | 0.485 | -0.806 | 0.420 | [-1.344 – 0.561] |
| **Adverse events [ref = No]** |  |  |  |  |  |
| Event at T0 | -6.007 | 1.417 | -4.239 | < .001 | [-8.790 – -3.223] |
| Event at T1 | -3.693 | 1.895 | -1.949 | 0.052 | [-7.416 – 0.030] |
| Event at T2 | -2.762 | 1.840 | -1.501 | 0.134 | [-6.376 – 0.852] |
| **Positive events [ref = No]** |  |  |  |  |  |
| Event at T0 | -0.496 | 1.538 | -0.322 | 0.747 | [-3.517 – 2.526] |
| Event at T1 | 0.455 | 3.449 | 0.132 | 0.895 | [-6.320 – 7.231] |
| Event at T2 | 1.049 | 4.932 | 0.213 | 0.832 | [-8.639 – 10.737] |
| **Covariance parameters** | **Estimate** | **se** | **Wald Z** | **p-value** | **95% CI** |
| Between variance | 221.603 | 16.072 | 13.788 | <.001 | [192.239 – 255.452] |
| Within variance | 73.308 | 5.520 | 13.281 | <.001 | [63.250 – 84.966] |
| Intraclass correlation | 0.751 | - | - | - | - |

| **Table A7b.** Results of the test-retest analysis: mixed models for CPHQ social acceptance | | | | | |
| --- | --- | --- | --- | --- | --- |
| **Effect** | **Estimate** | **se** | **t** | **p-value** | **95% CI** |
| **Intercept** | 77.357 | 0.924 | 83.752 | < .001 | [75.543 – 79.171] |
| **Time [ref = T0]** |  |  |  |  |  |
| T1 | -0.844 | 0.416 | -2.030 | 0.043 | [-1.661 – -0.027] |
| T2 | -0.061 | 0.393 | -0.156 | 0.876 | [-0.832 – 0.709] |
| **Adverse events [ref = No]** |  |  |  |  |  |
| Event at T0 | -2.416 | 1.240 | -1.949 | 0.052 | [-4.852 – 0.019] |
| Event at T1 | -2.259 | 1.658 | -1.363 | 0.174 | [-5.517 – 0.998] |
| Event at T2 | -1.618 | 1.610 | -1.005 | 0.315 | [-4.780 – 1.545] |
| **Positive events [ref = No]** |  |  |  |  |  |
| Event at T0 | -0.739 | 1.346 | -0.549 | 0.583 | [-3.382 – 1.905] |
| Event at T1 | 0.983 | 3.018 | 0.326 | 0.745 | [-4.945 – 6.912] |
| Event at T2 | 0.214 | 4.315 | 0.050 | 0.960 | [-8.262 – 8.691] |
| **Covariance parameters** | **Estimate** | **se** | **Wald Z** | **p-value** | **95% CI** |
| Between variance | 173.116 | 11.992 | 14.436 | < .001 | [151.137 – 198.291] |
| Within variance | 47.643 | 3.430 | 13.890 | < .001 | [41.373 – 54.863] |
| Intraclass correlation | 0.784 | - | - | - | - |

| **Table A7c.** Results of the test-retest analysis: mixed models for CPHQ wellbeing | | | | | |
| --- | --- | --- | --- | --- | --- |
| **Effect** | **Estimate** | **se** | **t** | **p-value** | **95% CI** |
| **Intercept** | 76.070 | 0.980 | 77.627 | < .001 | [74.145 – 77.994] |
| **Time [ref = T0]** |  |  |  |  |  |
| T1 | -0.127 | 0.369 | -0.343 | 0.732 | [-0.852 – 0.599] |
| T2 | -0.219 | 0.376 | -0.582 | 0.561 | [-0.957 – 0.519] |
| **Adverse events [ref = No]** |  |  |  |  |  |
| Event at T0 | -3.066 | 1.325 | -2.314 | 0.021 | [-5.670 – -0.463] |
| Event at T1 | -0.972 | 1.773 | -0.548 | 0.584 | [-4.454 – 2.510] |
| Event at T2 | -3.316 | 1.721 | -1.927 | 0.055 | [-6.696 – 0.065] |
| **Positive events [ref = No]** |  |  |  |  |  |
| Event at T0 | 0.679 | 1.439 | 0.472 | 0.637 | [-2.147 – 3.506] |
| Event at T1 | 1.941 | 3.226 | 0.602 | 0.548 | [-4.396 – 8.278] |
| Event at T2 | 5.090 | 4.613 | 1.104 | 0.270 | [-3.971 – 14.152] |
| **Covariance parameters** | **Estimate** | **se** | **Wald Z** | **p-value** | **95% CI** |
| Between variance | 207.242 | 13.461 | 15.396 | < .001 | [182.470 – 235.377] |
| Within variance | 37.077 | 2.122 | 17.472 | < .001 | [33.143 – 41.478] |
| Intraclass correlation | 0.848 | - | - | - | - |

| **Table A7d.** Results of the test-retest analysis: mixed models for CPHQ vitality | | | | | |
| --- | --- | --- | --- | --- | --- |
| **Effect** | **Estimate** | **se** | **t** | **p-value** | **95% CI** |
| **Intercept** | 69.979 | 1.194 | 58.632 | < .001 | [67.635 – 72.323] |
| **Time [ref = T0]** |  |  |  |  |  |
| T1 | -0.161 | 0.538 | -0.300 | 0.765 | [-1.218 – 0.896] |
| T2 | -0.829 | 0.491 | -1.687 | 0.092 | [-1.793 – 0.135] |
| **Adverse events [ref = No]** |  |  |  |  |  |
| Event at T0 | -5.861 | 1.604 | -3.655 | < .001 | [-9.012 – -2.711] |
| Event at T1 | -3.846 | 2.145 | -1.793 | 0.074 | [-8.061 – 0.368] |
| Event at T2 | -5.391 | 2.083 | -2.588 | 0.010 | [-9.483 – -1.300] |
| **Positive events [ref = No]** |  |  |  |  |  |
| Event at T0 | 1.886 | 1.741 | 1.083 | 0.279 | [-1.534 – 5.307] |
| Event at T1 | 2.132 | 3.905 | 0.546 | 0.585 | [-5.538 – 9.802] |
| Event at T2 | 7.256 | 5.583 | 1.300 | 0.194 | [-3.710 – 18.222] |
| **Covariance parameters** | **Estimate** | **se** | **Wald Z** | **p-value** | **95% CI** |
| Between variance | 285.684 | 20.398 | 14.009 | < .001 | [248.448 – 328.667] |
| Within variance | 81.857 | 6.808 | 12.024 | < .001 | [69.544 – 96.350] |
| Intraclass correlation | 0.777 | - | - | - | - |

| **Table A7e.** Results of the test-retest analysis: mixed models for CPHQ social support | | | | | |
| --- | --- | --- | --- | --- | --- |
| **Effect** | **Estimate** | **se** | **t** | **p-value** | **95% CI** |
| **Intercept** | 75.343 | 0.874 | 86.253 | < .001 | [73.628 – 77.059] |
| **Time [ref = T0]** |  |  |  |  |  |
| T1 | -0.476 | 0.419 | -1.136 | 0.257 | [-1.299 – 0.347] |
| T2 | -0.338 | 0.365 | -0.925 | 0.355 | [-1.054 – 0.379] |
| **Adverse events [ref = No]** |  |  |  |  |  |
| Event at T0 | 0.012 | 1.171 | 0.010 | 0.992 | [-2.288 – 2.312] |
| Event at T1 | 0.002 | 1.566 | 0.001 | 0.999 | [-3.075 – 3.079] |
| Event at T2 | -2.814 | 1.521 | -1.850 | 0.065 | [-5.801 – 0.173] |
| **Positive events [ref = No]** |  |  |  |  |  |
| Event at T0 | 2.363 | 1.271 | 1.859 | 0.064 | [-0.135 – 4.860] |
| Event at T1 | 2.933 | 2.851 | 1.029 | 0.304 | [-2.667 – 8.532] |
| Event at T2 | -0.477 | 4.076 | -0.117 | 0.907 | [-8.484 – 7.529] |
| **Covariance parameters** | **Estimate** | **se** | **Wald Z** | **p-value** | **95% CI** |
| Between variance | 145.020 | 11.468 | 12.646 | < .001 | [124.198 – 169.332] |
| Within variance | 52.958 | 5.443 | 9.729 | < .001 | [43.296 – 64.778] |
| Intraclass correlation | 0.733 | - | - | - | - |

| **Table A7f.** Results of the test-retest analysis: mixed models for CPHQ financial resources | | | | | |
| --- | --- | --- | --- | --- | --- |
| **Effect** | **Estimate** | **se** | **t** | **p-value** | **95% CI** |
| **Intercept** | 79.318 | 1.061 | 74.748 | < .001 | [77.234 – 81.402] |
| **Time [ref = T0]** |  |  |  |  |  |
| T1 | -0.622 | 0.495 | -1.256 | 0.210 | [-1.594 – 0.351] |
| T2 | -0.737 | 0.448 | -1.644 | 0.101 | [-1.616 – 0.143] |
| **Adverse events [ref = No]** | 0b | 0 | . | . | . |
| Event at T0 |  |  |  |  |  |
| Event at T1 | -1.016 | 1.423 | -0.714 | 0.476 | [-3.811 – 1.780] |
| Event at T2 | 0.298 | 1.904 | 0.156 | 0.876 | [-3.442 – 4.037] |
| **Positive events [ref = No]** | -2.281 | 1.848 | -1.234 | 0.218 | [-5.911 – 1.350] |
| Event at T0 |  |  |  |  |  |
| Event at T1 | 1.715 | 1.545 | 1.110 | 0.268 | [-1.321 – 4.750] |
| Event at T2 | 0.346 | 3.465 | 0.100 | 0.920 | [-6.460 – 7.152] |
| **Covariance parameters** | **Estimate** | **se** | **Wald Z** | **p-value** | **95% CI** |
| Between variance | 211.926 | 16.191 | 13.706 | < .001 | [192.356 – 256.041] |
| Within variance | 69.907 | 5.984 | 11.862 | < .001 | [59.110 – 82.677] |
| Intraclass correlation | 0.752 | - | - | - | - |

| **Table A7g.** Results of the test-retest analysis: mixed models for CPHQ health literacy | | | | | |
| --- | --- | --- | --- | --- | --- |
| **Effect** | **Estimate** | **se** | **t** | **p-value** | **95% CI** |
| **Intercept** | 80.363 | 0.732 | 109.763 | < .001 | [78.926 – 81.801] |
| **Time [ref = T0]** |  |  |  |  |  |
| T1 | -0.714 | 0.460 | -1.551 | 0.122 | [-1.618 – 0.190] |
| T2 | -0.391 | 0.462 | -0.848 | 0.397 | [-1.297 – 0.514] |
| **Adverse events [ref = No]** |  |  |  |  |  |
| Event at T0 | 0.423 | 0.946 | 0.447 | 0.655 | [-1.435 – 2.280] |
| Event at T1 | 0.531 | 1.265 | 0.420 | 0.675 | [-1.954 – 3.015] |
| Event at T2 | -0.106 | 1.228 | -0.086 | 0.932 | [-2.518 – 2.307] |
| **Positive events [ref = No]** |  |  |  |  |  |
| Event at T0 | 1.506 | 1.027 | 1.467 | 0.143 | [-0.511 – 3.522] |
| Event at T1 | -1.931 | 2.302 | -0.839 | 0.402 | [-6.453 – 2.591] |
| Event at T2 | -0.253 | 3.291 | -0.077 | 0.939 | [-6.718 – 6.212] |
| **Covariance parameters** | **Estimate** | **se** | **Wald Z** | **p-value** | **95% CI** |
| Between variance | 92.485 | 7.228 | 12.796 | < .001 | [79.351 – 107.794] |
| Within variance | 57.509 | 3.381 | 17.010 | < .001 | [51.250 – 64.532] |
| Intraclass correlation | 0.617 | - | - | - | - |

| **Table A7h.** Results of the test-retest analysis: mixed models for CPHQ mobility | | | | | |
| --- | --- | --- | --- | --- | --- |
| **Effect** | **Estimate** | **se** | **t** | **p-value** | **95% CI** |
| **Intercept** | 77.870 | 0.863 | 90.196 | < .001 | [76.175 – 79.565] |
| **Time [ref = T0]** |  |  |  |  |  |
| T1 | -0.322 | 0.456 | -0.707 | 0.480 | [-1.217 – 0.573] |
| T2 | -0.691 | 0.467 | -1.479 | 0.139 | [-1.607 – 0.226] |
| **Adverse events [ref = No]** |  |  |  |  |  |
| Event at T0 | -1.958 | 1.138 | -1.720 | 0.086 | [-4.193 – 0.278] |
| Event at T1 | -0.487 | 1.522 | -0.320 | 0.749 | [-3.478 – 2.503] |
| Event at T2 | -1.668 | 1.478 | -1.129 | 0.259 | [-4.572 – 1.235] |
| **Positive events [ref = No]** |  |  |  |  |  |
| Event at T0 | 0.670 | 1.236 | 0.543 | 0.588 | [-1.757 – 3.097] |
| Event at T1 | 1.636 | 2.770 | 0.591 | 0.555 | [-3.806 – 7.079] |
| Event at T2 | 4.772 | 3.961 | 1.205 | 0.229 | [-3.009 – 12.553] |
| **Covariance parameters** | **Estimate** | **se** | **Wald Z** | **p-value** | **95% CI** |
| Between variance | 143.864 | 10.067 | 14.290 | < .001 | [125.426 – 165.012] |
| Within variance | 56.503 | 3.126 | 18.073 | < .001 | [50.696 – 62.975] |
| Intraclass correlation | 0.718 | - | - | - | - |

| **Table A7i.** Results of the test-retest analysis: mixed models for CPHQ total score | | | | | |
| --- | --- | --- | --- | --- | --- |
| **Effect** | **Estimate** | **se** | **t** | **p-value** | **95% CI** |
| **Intercept** | 76.348 | 0.713 | 107.096 | < .001 | [74.947 – 77.748] |
| **Time [ref = T0]** |  |  |  |  |  |
| T1 | -0.431 | 0.240 | -1.796 | 0.073 | [-0.903 – 0.040] |
| T2 | -0.457 | 0.234 | -1.956 | 0.051 | [-0.916 – 0.002] |
| **Adverse events [ref = No]** |  |  |  |  |  |
| Event at T0 | -2.496 | 0.970 | -2.573 | 0.010 | [-4.402 – -0.590] |
| Event at T1 | -1.300 | 1.298 | -1.002 | 0.317 | [-3.849 – 1.249] |
| Event at T2 | -2.483 | 1.260 | -1.971 | 0.049 | [-4.958 – -0.008] |
| **Positive events [ref = No]** |  |  |  |  |  |
| Event at T0 | 0.944 | 1.053 | 0.896 | 0.371 | [-1.125 – 3.013] |
| Event at T1 | 1.002 | 2.362 | 0.424 | 0.671 | [-3.637 – 5.642] |
| Event at T2 | 2.260 | 3.377 | 0.669 | 0.504 | [-4.373 – 8.894] |
| **Covariance parameters** | **Estimate** | **se** | **Wald Z** | **p-value** | **95% CI** |
| Between variance | 111.754 | 7.212 | 15.496 | < .001 | [98.476 – 126.852] |
| Within variance | 15.697 | 1.030 | 15.233 | < .001 | [13.802 – 17.852] |
| Intraclass correlation | 0.877 | - | - | - | - |

| **Table A8.** Results of the test-retest analyses: weighted Kappa analyses (n=543) | | | | | | |
| --- | --- | --- | --- | --- | --- | --- |
|  | **T0 vs T1** | | | **T0 vs T2** | | |
| **Domain** | **Kappa [95% CI]** | **Z** | **p-value** | **Kappa [95% CI]** | **Z** | **p-value** |
| Mental relaxation | 0.759 [0.714-0.804] | 17.690 | <0.001 | 0.709[0.661-0.758] | 16.533 | <0.001 |
| Wellbeing | 0.767 [0.722-0.812] | 17.879 | <0.001 | 0.802 [0.765-0.839] | 18.683 | <0.001 |
| Mobility | 0.671 [0.616-0.726] | 15.688 | <0.001 | 0.667 [0.612-0.721] | 15.546 | <0.001 |
| Vitality | 0.810 [0.774-0.845] | 18.885 | <0.001 | 0.752 [0.706-0.798] | 17.586 | <0.001 |
| Social acceptance | 0.692 [0.644-0.740] | 16.166 | <0.001 | 0.692 [0.642-0.742] | 16.124 | <0.001 |
| Social support | 0.721 [0.671-0.771] | 16.815 | <0.001 | 0.611 [0.557-0.666] | 14.788 | <0.001 |
| Financial resources | 0.731 [0.679-0.783] | 17.124 | <0.001 | 0.686 [0.629-0.742] | 16.105 | <0.001 |
| Health literacy | 0.621 [0.563-0.679] | 14.495 | <0.001 | 0.603 [0.544-0.662] | 14.105 | <0.001 |
| Total score | 0.795 [0.758-0.831] | 18.541 | <0.001 | 0.790 [0.755-0.825] | 18.433 | <0.001 |

| **Table A9a**. Complete item intercorrelation for subsample T1 (N=714) | | | | | | | | | | | | | | | | | | | | |
| --- | --- | --- | --- | --- | --- | --- | --- | --- | --- | --- | --- | --- | --- | --- | --- | --- | --- | --- | --- | --- |
|  | **Mental Relaxation** | | **Social Acceptance** | | | **Wellbeing** | | | | **Vitality** | | **Social Support** | | | **Financial**  **Resources** | | **Health Literacy** | | **Mobility** | |
|  | i1 | i2 | i3 | i4 | i5 | i6 | i7 | i8 | i9 | i10 | i11 | i12 | i13 | i14 | i15 | i16 | i17 | i18 | i19 | i20 |
| i1 | 1 | 0.69*** | 0.40*** | 0.41*** | 0.39*** | 0.56*** | 0.50*** | 0.44*** | 0.57*** | 0.48*** | 0.44*** | 0.24*** | 0.31*** | 0.26*** | 0.25*** | 0.24*** | 0.17*** | 0.33*** | 0.32*** | 0.34*** |
| i2 | 0.69*** | 1 | 0.41*** | 0.37*** | 0.39*** | 0.53*** | 0.43*** | 0.43*** | 0.59*** | 0.44*** | 0.42*** | 0.28*** | 0.32*** | 0.26*** | 0.25*** | 0.24*** | 0.18*** | 0.33*** | 0.36*** | 0.3*** |
| i3 | 0.40*** | 0.41*** | 1 | 0.69*** | 0.61*** | 0.52*** | 0.51*** | 0.53*** | 0.48*** | 0.38*** | 0.37*** | 0.49*** | 0.61*** | 0.45*** | 0.36*** | 0.37*** | 0.27*** | 0.40*** | 0.32*** | 0.38*** |
| i4 | 0.41*** | 0.37*** | 0.69*** | 1 | 0.64*** | 0.52*** | 0.57*** | 0.53*** | 0.51*** | 0.33*** | 0.35*** | 0.53*** | 0.69*** | 0.55*** | 0.31*** | 0.31*** | 0.24*** | 0.39*** | 0.29*** | 0.38*** |
| i5 | 0.39*** | 0.39*** | 0.61*** | 0.64*** | 1 | 0.46*** | 0.46*** | 0.46*** | 0.46*** | 0.28*** | 0.27*** | 0.45*** | 0.52*** | 0.43*** | 0.27*** | 0.26*** | 0.17*** | 0.34*** | 0.25*** | 0.25*** |
| i6 | 0.56*** | 0.53*** | 0.52*** | 0.52*** | 0.46*** | 1 | 0.69*** | 0.64*** | 0.64*** | 0.52*** | 0.51*** | 0.45*** | 0.52*** | 0.46*** | 0.34*** | 0.34*** | 0.22*** | 0.40*** | 0.35*** | 0.36*** |
| i7 | 0.50*** | 0.43*** | 0.51*** | 0.57*** | 0.46*** | 0.69*** | 1 | 0.67*** | 0.59*** | 0.43*** | 0.47*** | 0.44*** | 0.50*** | 0.46*** | 0.31*** | 0.31*** | 0.24*** | 0.39*** | 0.33*** | 0.40*** |
| i8 | 0.44*** | 0.43*** | 0.53*** | 0.53*** | 0.46*** | 0.64*** | 0.67*** | 1 | 0.59*** | 0.40*** | 0.40*** | 0.43*** | 0.51*** | 0.45*** | 0.29*** | 0.30*** | 0.23*** | 0.36*** | 0.3*** | 0.3*** |
| i9 | 0.57*** | 0.59*** | 0.48*** | 0.51*** | 0.46*** | 0.64*** | 0.59*** | 0.59*** | 1 | 0.46*** | 0.48*** | 0.46*** | 0.46*** | 0.46*** | 0.31*** | 0.29*** | 0.29*** | 0.42*** | 0.46*** | 0.43*** |
| i10 | 0.48*** | 0.44*** | 0.38*** | 0.33*** | 0.28*** | 0.52*** | 0.43*** | 0.40*** | 0.46*** | 1 | 0.78*** | 0.24*** | 0.3*** | 0.3*** | 0.35*** | 0.34*** | 0.18*** | 0.39*** | 0.57*** | 0.49*** |
| i11 | 0.44*** | 0.42*** | 0.37*** | 0.35*** | 0.27*** | 0.51*** | 0.47*** | 0.40*** | 0.48*** | 0.78*** | 1 | 0.27*** | 0.30*** | 0.34*** | 0.39*** | 0.36*** | 0.23*** | 0.42*** | 0.55*** | 0.51*** |
| i12 | 0.24*** | 0.28*** | 0.49*** | 0.53*** | 0.45*** | 0.45*** | 0.44*** | 0.43*** | 0.46*** | 0.24*** | 0.27*** | 1 | 0.75*** | 0.66*** | 0.32*** | 0.29*** | 0.29*** | 0.40*** | 0.27*** | 0.38*** |
| i13 | 0.31*** | 0.32*** | 0.61*** | 0.69*** | 0.52*** | 0.52*** | 0.5*** | 0.51*** | 0.46*** | 0.30*** | 0.30*** | 0.75*** | 1 | 0.59*** | 0.32*** | 0.32*** | 0.24*** | 0.36*** | 0.25*** | 0.36*** |
| i14 | 0.26*** | 0.26*** | 0.45*** | 0.55*** | 0.43*** | 0.46*** | 0.46*** | 0.45*** | 0.46*** | 0.30*** | 0.34*** | 0.66*** | 0.59*** | 1 | 0.35*** | 0.34*** | 0.26*** | 0.37*** | 0.3*** | 0.40*** |
| i15 | 0.25*** | 0.25*** | 0.36*** | 0.31*** | 0.27*** | 0.34*** | 0.31*** | 0.29*** | 0.31*** | 0.35*** | 0.39*** | 0.32*** | 0.32*** | 0.35*** | 1 | 0.82*** | 0.31*** | 0.38*** | 0.31*** | 0.35*** |
| i16 | 0.24*** | 0.24*** | 0.37*** | 0.31*** | 0.26*** | 0.34*** | 0.31*** | 0.30*** | 0.29*** | 0.34*** | 0.36*** | 0.29*** | 0.32*** | 0.34*** | 0.82*** | 1 | 0.38*** | 0.37*** | 0.31*** | 0.34*** |
| i17 | 0.17*** | 0.18*** | 0.27*** | 0.24*** | 0.17*** | 0.22*** | 0.24*** | 0.23*** | 0.29*** | 0.18*** | 0.23*** | 0.29*** | 0.24*** | 0.26*** | 0.31*** | 0.38*** | 1 | 0.43*** | 0.28*** | 0.27*** |
| i18 | 0.33*** | 0.33*** | 0.40*** | 0.39*** | 0.34*** | 0.40*** | 0.39*** | 0.36*** | 0.42*** | 0.39*** | 0.42*** | 0.4*** | 0.36*** | 0.37*** | 0.38*** | 0.37*** | 0.43*** | 1 | 0.46*** | 0.45*** |
| i19 | 0.32*** | 0.36*** | 0.32*** | 0.29*** | 0.25*** | 0.35*** | 0.33*** | 0.30*** | 0.46*** | 0.57*** | 0.55*** | 0.27*** | 0.25*** | 0.30*** | 0.31*** | 0.31*** | 0.28*** | 0.46*** | 1 | 0.55*** |
| i20 | 0.34*** | 0.30*** | 0.38*** | 0.38*** | 0.25*** | 0.36*** | 0.40*** | 0.30*** | 0.43*** | 0.49*** | 0.51*** | 0.38*** | 0.36*** | 0.40*** | 0.35*** | 0.34*** | 0.27*** | 0.45*** | 0.55*** | 1 |
| *Note.* i1=I feel calm; i2=I can relax; i3=I feel accepted; i4=I feel that I belong; i5=I feel excluded; i6=I feel happy; i7=I feel my life is meaningful; i8=I feel grateful for my life; i9=I can enjoy; i10=I feel fit; i11=I feel healthy; i12=I have people who support me; i13=I feel that people support me; i14=I have people with I can do fun things with; i15=I have money to do the things that I find important; i16=I have money to live a healthy life; i17=I can understand information about my health; i18=I can find help for my health; i19=I can move; i20=I can be active in my environment; *p<0.05; **p<0.01; ***p<0.001. | | | | | | | | | | | | | | | | | | | | |

| **Table A9b**. Complete item intercorrelation for subsample T2 (N=543) | | | | | | | | | | | | | | | | | | | | |
| --- | --- | --- | --- | --- | --- | --- | --- | --- | --- | --- | --- | --- | --- | --- | --- | --- | --- | --- | --- | --- |
|  | **Mental Relaxation** | | **Social Acceptance** | | | **Wellbeing** | | | | **Vitality** | | **Social Support** | | | **Financial**  **Resources** | | **Health Literacy** | | **Mobility** | |
|  | i1 | i2 | i3 | i4 | i5 | i6 | i7 | i8 | i9 | i10 | i11 | i12 | i13 | i14 | i15 | i16 | i17 | i18 | i19 | i20 |
| i1 | 1 | 0.68*** | 0.42*** | 0.43*** | 0.41*** | 0.6*** | 0.46*** | 0.44*** | 0.54*** | 0.50*** | 0.49*** | 0.31*** | 0.34*** | 0.3*** | 0.29*** | 0.25*** | 0.18*** | 0.3*** | 0.32*** | 0.29*** |
| i2 | 0.68*** | 1 | 0.43*** | 0.43*** | 0.43*** | 0.55*** | 0.44*** | 0.42*** | 0.61*** | 0.50*** | 0.47*** | 0.33*** | 0.35*** | 0.31*** | 0.29*** | 0.21*** | 0.21*** | 0.3*** | 0.34*** | 0.31*** |
| i3 | 0.42*** | 0.43*** | 1 | 0.68*** | 0.62*** | 0.54*** | 0.49*** | 0.49*** | 0.50*** | 0.42*** | 0.42*** | 0.52*** | 0.57*** | 0.51*** | 0.38*** | 0.39*** | 0.31*** | 0.41*** | 0.34*** | 0.38*** |
| i4 | 0.43*** | 0.43*** | 0.68*** | 1 | 0.64*** | 0.55*** | 0.56*** | 0.52*** | 0.52*** | 0.42*** | 0.4*** | 0.58*** | 0.70*** | 0.59*** | 0.39*** | 0.37*** | 0.23*** | 0.44*** | 0.33*** | 0.42*** |
| i5 | 0.41*** | 0.43*** | 0.62*** | 0.64*** | 1 | 0.53*** | 0.47*** | 0.47*** | 0.46*** | 0.40*** | 0.39*** | 0.43*** | 0.49*** | 0.45*** | 0.28*** | 0.29*** | 0.22*** | 0.35*** | 0.30*** | 0.33*** |
| i6 | 0.6*** | 0.55*** | 0.54*** | 0.55*** | 0.53*** | 1 | 0.70*** | 0.66*** | 0.68*** | 0.55*** | 0.58*** | 0.49*** | 0.51*** | 0.48*** | 0.38*** | 0.36*** | 0.24*** | 0.41*** | 0.40*** | 0.37*** |
| i7 | 0.46*** | 0.44*** | 0.49*** | 0.56*** | 0.47*** | 0.70*** | 1 | 0.67*** | 0.60*** | 0.48*** | 0.48*** | 0.46*** | 0.50*** | 0.46*** | 0.34*** | 0.31*** | 0.24*** | 0.40*** | 0.33*** | 0.36*** |
| i8 | 0.44*** | 0.42*** | 0.49*** | 0.52*** | 0.47*** | 0.66*** | 0.67*** | 1 | 0.60*** | 0.43*** | 0.45*** | 0.43*** | 0.47*** | 0.44*** | 0.30*** | 0.31*** | 0.25*** | 0.41*** | 0.34*** | 0.39*** |
| i9 | 0.54*** | 0.61*** | 0.50*** | 0.52*** | 0.46*** | 0.68*** | 0.6*** | 0.60*** | 1 | 0.49*** | 0.50*** | 0.43*** | 0.46*** | 0.44*** | 0.33*** | 0.29*** | 0.30*** | 0.4*** | 0.41*** | 0.42*** |
| i10 | 0.5*** | 0.50*** | 0.42*** | 0.42*** | 0.4*** | 0.55*** | 0.48*** | 0.43*** | 0.49*** | 1 | 0.77*** | 0.32*** | 0.34*** | 0.38*** | 0.34*** | 0.33*** | 0.2*** | 0.36*** | 0.58*** | 0.46*** |
| i11 | 0.49*** | 0.47*** | 0.42*** | 0.40*** | 0.39*** | 0.58*** | 0.48*** | 0.45*** | 0.50*** | 0.77*** | 1 | 0.31*** | 0.35*** | 0.37*** | 0.37*** | 0.38*** | 0.23*** | 0.33*** | 0.55*** | 0.46*** |
| i12 | 0.31*** | 0.33*** | 0.52*** | 0.58*** | 0.43*** | 0.49*** | 0.46*** | 0.43*** | 0.43*** | 0.32*** | 0.31*** | 1 | 0.74*** | 0.64*** | 0.37*** | 0.33*** | 0.26*** | 0.48*** | 0.36*** | 0.45*** |
| i13 | 0.34*** | 0.35*** | 0.57*** | 0.70*** | 0.49*** | 0.51*** | 0.50*** | 0.47*** | 0.46*** | 0.34*** | 0.35*** | 0.74*** | 1 | 0.62*** | 0.36*** | 0.35*** | 0.29*** | 0.44*** | 0.30*** | 0.40*** |
| i14 | 0.3*** | 0.31*** | 0.51*** | 0.59*** | 0.45*** | 0.48*** | 0.46*** | 0.44*** | 0.44*** | 0.38*** | 0.37*** | 0.64*** | 0.62*** | 1 | 0.34*** | 0.33*** | 0.24*** | 0.37*** | 0.36*** | 0.40*** |
| i15 | 0.29*** | 0.29*** | 0.38*** | 0.39*** | 0.28*** | 0.38*** | 0.34*** | 0.30*** | 0.33*** | 0.34*** | 0.37*** | 0.37*** | 0.36*** | 0.34*** | 1 | 0.78*** | 0.28*** | 0.35*** | 0.31*** | 0.34*** |
| i16 | 0.25*** | 0.21*** | 0.39*** | 0.37*** | 0.29*** | 0.36*** | 0.31*** | 0.31*** | 0.29*** | 0.33*** | 0.38*** | 0.33*** | 0.35*** | 0.33*** | 0.78*** | 1 | 0.33*** | 0.32*** | 0.33*** | 0.29*** |
| i17 | 0.18*** | 0.21*** | 0.31*** | 0.23*** | 0.22*** | 0.24*** | 0.24*** | 0.25*** | 0.30*** | 0.20*** | 0.23*** | 0.26*** | 0.29*** | 0.24*** | 0.28*** | 0.33*** | 1 | 0.45*** | 0.25*** | 0.31*** |
| i18 | 0.30*** | 0.30*** | 0.41*** | 0.44*** | 0.35*** | 0.41*** | 0.40*** | 0.41*** | 0.40*** | 0.36*** | 0.33*** | 0.48*** | 0.44*** | 0.37*** | 0.35*** | 0.32*** | 0.45*** | 1 | 0.35*** | 0.44*** |
| i19 | 0.32*** | 0.34*** | 0.34*** | 0.33*** | 0.3*** | 0.40*** | 0.33*** | 0.34*** | 0.41*** | 0.58*** | 0.55*** | 0.36*** | 0.30*** | 0.36*** | 0.31*** | 0.33*** | 0.25*** | 0.35*** | 1 | 0.5*** |
| i20 | 0.29*** | 0.31*** | 0.38*** | 0.42*** | 0.33*** | 0.37*** | 0.36*** | 0.39*** | 0.42*** | 0.46*** | 0.46*** | 0.45*** | 0.4*** | 0.40*** | 0.34*** | 0.29*** | 0.31*** | 0.44*** | 0.5*** | 1 |
| *Note.* i1=I feel calm; i2=I can relax; i3=I feel accepted; i4=I feel that I belong; i5=I feel excluded; i6=I feel happy; i7=I feel my life is meaningful; i8=I feel grateful for my life; i9=I can enjoy; i10=I feel fit; i11=I feel healthy; i12=I have people who support me; i13=I feel that people support me; i14=I have people with I can do fun things with; i15=I have money to do the things that I find important; i16=I have money to live a healthy life; i17=I can understand information about my health; i18=I can find help for my health; i19=I can move; i20=I can be active in my environment; *p<0.05; **p<0.01; ***p<0.001. | | | | | | | | | | | | | | | | | | | | |
