# Supplementary material for: A longitudinal psychometric evaluation of a context-sensitive positive health questionnaire for measuring broad health in Dutch adults
Source: J Patient Rep Outcomes. 2026 Jun 2;10:136. doi: 10.1186/s41687-026-01098-x (PMC13454085; doi:10.1186/s41687-026-01098-x)
Supplement: Supplementary file 2 [file 41687_2026_1098_MOESM2_ESM.docx]

**Appendix X: Overview of Hypotheses Concerning the CPHQ2.0**

**Part 1: Hypotheses concerning concurrent validity**

The following hypotheses were formulated based on the conceptual alignment of the CPHQ2.0 domains with the Positive Health framework and the Capability Approach. Each domain was linked to external validation instruments that capture related constructs of health, well-being, and capability.

| **CPHQ2.0 domain** | **Expected correlation with subscale** | **Rationale** |
| --- | --- | --- |
| **Wellbeing** | PH-17 Contentment; PWI Life Satisfaction; ICECAP-A Wellbeing | Reflects overall sense of wellbeing and life satisfaction. |
| **Vitality** | PH-17 Physical Fitness; PWI Health | Reflects perceived (physical) health status. |
| **Mobility** | EQ-5D-5L Mobility; EQ-5D-5L Usual Activities; PH-17 Physical Fitness | Reflects ability to move and perform daily activities. |
| **Social Support** | PH-17 Social Relations; PWI Relationships | Reflects availability and quality of close social relationships. |
| **Social Acceptance** | PWI Community Connectedness; PH-17 Social Relations | Reflects sense of belonging and inclusion in social networks. |
| **Financial Resources** | PWI Standard of Living | Reflects material and financial security for daily living. |
| **Mental Relaxation** | EQ-5D-5L Anxiety/Depression; PH-17 Mental Functioning; BRS Resilience | Reflects emotional stability, absence of distress, and coping ability. |
| **Health Literacy** | PH-17 Daily Life Management (expected relative weak) | Reflects ability to manage daily life and health-related tasks. |
| **Total Score** | PH-17 Total; PWI Total; ICECAP-A Total | Reflects overall health, wellbeing, and capability across multiple domains. |

**Part 2: Hypotheses concerning subgroup analyses**

**Hypothesis 1: Gender**

- **Expectation**: Men will score higher (=better health) on the CPHQ2.0 compared to women.
- **Supporting Evidence**:
  1. Data from the Health Survey (CBS) shows that men consistently report better self-rated health over multiple years.
     *Source*: [Self-rated health | Age and Gender | Public Health and Care (vzinfo.nl)](https://www.vzinfo.nl/ervaren-gezondheid/leeftijd-en-geslacht).
  2. Euroqol data indicates that men score slightly higher on the EQ-VAS (=better health) than women.
     *Source*: [Population Norms for the EQ-5D - NCBI Bookshelf (nih.gov)](https://www.ncbi.nlm.nih.gov/books/NBK500364/).

**Hypothesis 2: Age**

- **Expectations**:
  1. Adults of working age (18-64 years) will score higher (=better health) on the CPHQ2.0 compared to adults aged 65 and older.
  2. As participants age, scores will increase (=better health) on the CPHQ2.0 domains mental relaxation and enjoyment.
- **Supporting Evidence**:
  1. Older respondents report worse self-rated health according to the Health Survey (CBS).
     *Source*: [Self-rated health | Age and Gender | Public Health and Care (vzinfo.nl)](https://www.vzinfo.nl/ervaren-gezondheid/oorzaken-en-gevolgen).
  2. Euroqol data confirms that older adults score lower (=worse health) on the EQ-VAS compared to younger adults.
     *Source*: [Population Norms for the EQ-5D - NCBI Bookshelf (nih.gov)](https://www.ncbi.nlm.nih.gov/books/NBK500364/).
  3. Mental health indicators, such as calmness and happiness, improve with age.
     *Source*: [Mental health issues | Public Health and Care (vzinfo.nl)](https://www.vzinfo.nl/mentale-gezondheid/mentale-problemen).

**Hypothesis 3: Migration Background**

- **Expectation**: Participants with a migration background will score lower (=worse health) on the CPHQ2.0 compared to participants with a Dutch cultural background.
- **Supporting Evidence**:
  1. A 2021 study in England involving 1,394,361 participants found that migrants scored worse on the EQ5D-5L compared to individuals without a migration background.
     *Source*: [Ethnic inequalities in health-related quality of life - The Lancet Public Health](https://www.thelancet.com/article/S2468-2667(20)30287-5/fulltext).

**Hypothesis 4: Education Level, Income, and SES**

- **Expectation**: Participants with higher income, education levels, or socioeconomic status will score higher (=better health) on the CPHQ2.0 compared to those with lower income or education levels.
- **Supporting Evidence**:
  1. The Health Survey (CBS) shows that participants in higher education and income groups report better self-rated health.
     *Source*: [Self-rated health | Work and Income | Public Health and Care (vzinfo.nl)](https://www.vzinfo.nl/ervaren-gezondheid/werk-en-inkomen).
  2. Studies using ICECAP-A instruments in the Netherlands and Norway confirm these patterns.
     *Sources*:
     - [The ICECAP-A instrument for capabilities - PMC (nih.gov)](https://pmc.ncbi.nlm.nih.gov/articles/PMC8921020/).

**Hypothesis 5: Living Situation**

- **Expectation**: Participants who live together or are married will score higher (=better health) on the CPHQ2.0 compared to those who are single or live alone.
- **Supporting Evidence**:
  1. International studies have found differences in self-rated health/quality of life between married and single individuals.
     *Sources*:
     - [Socio-economic correlates of quality of life | Health and Quality of Life Outcomes](https://hqlo.biomedcentral.com/articles/10.1186/s12955-022-01966-2).
     - [Happy, Healthy and Wedded? | PMC (nih.gov)](https://pmc.ncbi.nlm.nih.gov/articles/PMC8888778/).
  2. Dutch research using the ICECAP-A instrument shows that participants in relationships score better than those without relationships.
     *Source*: [The ICECAP-A instrument for capabilities - PMC (nih.gov)](https://pmc.ncbi.nlm.nih.gov/articles/PMC8921020/).

**Hypothesis 6: Significant Life Events**

- **Expectations**:
  1. Participants who recently experienced life events (positive or negative) will score differently on the CPHQ2.0 compared to those who did not.
  2. There will be a cumulative effect of negative or positive life events on the CPHQ2.0 score.
- **Supporting Evidence**:
  1. A UK study (~1000 participants) found a cumulative relationship between life events and self-rated health, with negative events leading to worse outcomes and positive events leading to better outcomes.
     *Source*: [The impact of life events on health and well-being - BMC Research Notes](https://bmcresnotes.biomedcentral.com/articles/10.1186/s13104-016-2278-x).
  2. A US study confirmed temporary changes in health scores due to negative and positive life events.
     *Source*: [Exposure to negative life events - PubMed (nih.gov)](https://pubmed.ncbi.nlm.nih.gov/36989835/).
  3. Negative life events significantly impact mental well-being for both genders.
     *Source*: [Gender, life events, and mental well-being - PubMed (nih.gov)](https://pubmed.ncbi.nlm.nih.gov/37029493/).

**Hypothesis 7: Chronic Conditions**

- **Expectation**: Participants with one or more chronic conditions will score lower (=worse health) on the CPHQ2.0 compared to participants without chronic conditions.
- **Supporting Evidence**:
  1. The Health Survey (CBS) shows that participants with chronic diseases report worse self-rated health than those without such conditions.
     *Source*: [Self-rated health | Causes and Consequences | Public Health and Care (vzinfo.nl)](https://www.vzinfo.nl/ervaren-gezondheid/oorzaken-en-gevolgen).
