# Supplementary material for: A longitudinal psychometric evaluation of a context-sensitive positive health questionnaire for measuring broad health in Dutch adults
Source: J Patient Rep Outcomes. 2026 Jun 2;10:136. doi: 10.1186/s41687-026-01098-x (PMC13454085; doi:10.1186/s41687-026-01098-x)
Supplement: Supplementary file 3 [file 41687_2026_1098_MOESM3_ESM.docx]

**Appendix C**

- **Part 1.** IRT results for Mental Relaxation
- **Part 2.** IRT results for Social Acceptance
- **Part 3.** IRT results for Wellbeing
- **Part 4:** IRT results for Vitality
- **Part 5.** IRT results for Social Support
- **Part 6.** IRT results for Financial Resources
- **Part 7.** IRT results for Health Literacy
- **Part 8.** IRT results for Physical self-efficacy

**Part 1:** IRT results for Mental Relaxation

# 1. Model Fit Indices

| **Model** | **AIC** | **BIC** | **HQ** | **SABIC** | **logLik** | **chi_square** | **p_value** |
| --- | --- | --- | --- | --- | --- | --- | --- |
| GPCM | 1,944.219 | 1,986.365 | 1,960.757 | 1,954.624 | -962.109 | 11.115 | 0.677 |

# 2. Item Parameters

| **items.a** | **items.b1** | **items.b2** | **items.b3** | **items.b4** | **means** | **F1** | **Variable** |
| --- | --- | --- | --- | --- | --- | --- | --- |
| 3.174 | -1.351 | 0.586 | 1.625 | 3.050 | 0 | 1 | CPHQ2.0_28_feelcalm |
| 3.217 | -1.079 | 0.759 | 1.890 | 3.343 | 0 | 1 | CPHQ2.0_15_canrelax |

# 3. Person Score Distribution

| **Var1** | **Var2** | **Freq** |
| --- | --- | --- |
|  | F1 | Min. :-1.702228 |
|  | F1 | 1st Qu.:-0.204207 |
|  | F1 | Median :-0.204207 |
|  | F1 | Mean :-0.000087 |
|  | F1 | 3rd Qu.: 0.498391 |
|  | F1 | Max. : 3.231339 |

# 4. Item Loadings and Communalities

| **F1** | **h2** | **F1.1** | **Variable** |
| --- | --- | --- | --- |
| 0.881 | 0.777 | 1 | CPHQ2.0_28_feelcalm |
| 0.884 | 0.781 | 1 | CPHQ2.0_15_canrelax |

**Part 2:** IRT results for Social Acceptance

# 1. Model Fit Indices

| **Model** | **AIC** | **BIC** | **HQ** | **SABIC** | **logLik** | **chi_square** | **p_value** |
| --- | --- | --- | --- | --- | --- | --- | --- |
| GPCM | 2,413.689 | 2,472.693 | 2,436.842 | 2,428.256 | -1,192.844 | 102.056 | 0.1 |

# 2. Item Parameters

| **items.a** | **items.b1** | **items.b2** | **items.b3** | **items.b4** | **means** | **F1** | **Variable** |
| --- | --- | --- | --- | --- | --- | --- | --- |
| 3.570 | -0.779 | 1.455 | 2.432 |  | 0 | 1 | CPHQ2.0_8_feelaccepted |
| 4.845 | -1.056 | 0.878 | 2.162 | 3.145 | 0 | 1 | CPHQ2.0_10_feelbelonged |
| 2.225 | -0.538 | 1.097 | 2.421 | 3.039 | 0 | 1 | CPHQ2.0_7_feelexcluded_r |

# 3. Person Score Distribution

| **Var1** | **Var2** | **Freq** |
| --- | --- | --- |
|  | F1 | Min. :-1.598002 |
|  | F1 | 1st Qu.:-0.535993 |
|  | F1 | Median : 0.111997 |
|  | F1 | Mean :-0.000148 |
|  | F1 | 3rd Qu.: 0.517157 |
|  | F1 | Max. : 3.028700 |

# 4. Item Loadings and Communalities

| **F1** | **h2** | **F1.1** | **Variable** |
| --- | --- | --- | --- |
| 0.903 | 0.815 | 1 | CPHQ2.0_8_feelaccepted |
| 0.943 | 0.890 | 1 | CPHQ2.0_10_feelbelonged |
| 0.794 | 0.631 | 1 | CPHQ2.0_7_feelexcluded_r |

**Part 3:** IRT results for Wellbeing

# 1. Model Fit Indices

| **Model** | **AIC** | **BIC** | **HQ** | **SABIC** | **logLik** | **chi_square** | **p_value** |
| --- | --- | --- | --- | --- | --- | --- | --- |
| GPCM | 3,465.678 | 3,549.97 | 3,498.754 | 3,486.489 | -1,712.839 | 234.239 | 1 |

# 2. Item Parameters

| **items.a** | **items.b1** | **items.b2** | **items.b3** | **items.b4** | **means** | **F1** | **Variable** |
| --- | --- | --- | --- | --- | --- | --- | --- |
| 3.423 | -1.100 | 0.728 | 1.978 | 2.817 | 0 | 1 | CPHQ2.0_29_feelhappy |
| 2.815 | -1.202 | 0.762 | 2.001 | 3.204 | 0 | 1 | CPHQ2.0_30_lifehasmeaning |
| 1.841 | -0.631 | 1.354 | 2.332 | 3.452 | 0 | 1 | CPHQ2.0_11_feelgrateful |
| 2.272 | -0.723 | 1.328 | 3.040 | 2.770 | 0 | 1 | CPHQ2.0_18_canenjoy |

# 3. Person Score Distribution

| **Var1** | **Var2** | **Freq** |
| --- | --- | --- |
|  | F1 | Min. :-1.7820619 |
|  | F1 | 1st Qu.:-0.4808601 |
|  | F1 | Median : 0.0074044 |
|  | F1 | Mean :-0.0004801 |
|  | F1 | 3rd Qu.: 0.5464178 |
|  | F1 | Max. : 2.6457003 |

# 4. Item Loadings and Communalities

| **F1** | **h2** | **F1.1** | **Variable** |
| --- | --- | --- | --- |
| 0.895 | 0.802 | 1 | CPHQ2.0_29_feelhappy |
| 0.856 | 0.732 | 1 | CPHQ2.0_30_lifehasmeaning |
| 0.734 | 0.539 | 1 | CPHQ2.0_11_feelgrateful |
| 0.800 | 0.641 | 1 | CPHQ2.0_18_canenjoy |

**Part 4:** IRT results for Vitality

# 1. Model Fit Indices

| **Model** | **AIC** | **BIC** | **HQ** | **SABIC** | **logLik** | **chi_square** | **p_value** |
| --- | --- | --- | --- | --- | --- | --- | --- |
| GPCM | 1,926.449 | 1,968.595 | 1,942.987 | 1,936.855 | -953.225 | 1.509 | 1 |

# 2. Item Parameters

| **items.a** | **items.b1** | **items.b2** | **items.b3** | **items.b4** | **means** | **F1** | **Variable** |
| --- | --- | --- | --- | --- | --- | --- | --- |
| 5.096 | -1.302 | 0.261 | 1.211 | 2.394 | 0 | 1 | CPHQ2.0_26_feelfit |
| 5.655 | -1.203 | 0.575 | 1.443 | 2.540 | 0 | 1 | CPHQ2.0_25_feelhealthy |

# 3. Person Score Distribution

| **Var1** | **Var2** | **Freq** |
| --- | --- | --- |
|  | F1 | Min. :-1.7822179 |
|  | F1 | 1st Qu.:-0.3553742 |
|  | F1 | Median :-0.3553742 |
|  | F1 | Mean : 0.0008511 |
|  | F1 | 3rd Qu.: 0.8140937 |
|  | F1 | Max. : 2.7976878 |

# 4. Item Loadings and Communalities

| **F1** | **h2** | **F1.1** | **Variable** |
| --- | --- | --- | --- |
| 0.948 | 0.900 | 1 | CPHQ2.0_26_feelfit |
| 0.958 | 0.917 | 1 | CPHQ2.0_25_feelhealthy |

**Part 5:** IRT results for Social Support

# 1. Model Fit Indices

| **Model** | **AIC** | **BIC** | **HQ** | **SABIC** | **logLik** | **chi_square** | **p_value** |
| --- | --- | --- | --- | --- | --- | --- | --- |
| GPCM | 2,367.959 | 2,426.964 | 2,391.112 | 2,382.527 | -1,169.98 | 50.161 | 0.999 |

# 2. Item Parameters

| **items.a** | **items.b1** | **items.b2** | **items.b3** | **items.b4** | **means** | **F1** | **Variable** |
| --- | --- | --- | --- | --- | --- | --- | --- |
| 14.920 | -0.635 | 1.142 | 2.302 |  | 0 | 1 | CPHQ2.0_6_havesupport |
| 2.801 | -0.903 | 1.205 | 2.455 | 3.365 | 0 | 1 | CPHQ2.0_9_feelsupport |
| 1.815 | -1.190 | 1.277 | 2.892 | 2.948 | 0 | 1 | CPHQ2.0_1_havepeople |

# 3. Person Score Distribution

| **Var1** | **Var2** | **Freq** |
| --- | --- | --- |
|  | F1 | Min. :-1.564012 |
|  | F1 | 1st Qu.:-0.875902 |
|  | F1 | Median : 0.153930 |
|  | F1 | Mean :-0.000123 |
|  | F1 | 3rd Qu.: 0.502827 |
|  | F1 | Max. : 3.275377 |

# 4. Item Loadings and Communalities

| **F1** | **h2** | **F1.1** | **Variable** |
| --- | --- | --- | --- |
| 0.994 | 0.987 | 1 | CPHQ2.0_6_havesupport |
| 0.855 | 0.730 | 1 | CPHQ2.0_9_feelsupport |
| 0.730 | 0.532 | 1 | CPHQ2.0_1_havepeople |

**Part 6:** IRT results for Financial Resources

# 1. Model Fit Indices

| **Model** | **AIC** | **BIC** | **HQ** | **SABIC** | **logLik** | **chi_square** | **p_value** |
| --- | --- | --- | --- | --- | --- | --- | --- |
| GPCM | 1,625.467 | 1,667.613 | 1,642.005 | 1,635.873 | -802.734 | 8.533 | 0.86 |

# 2. Item Parameters

| **items.a** | **items.b1** | **items.b2** | **items.b3** | **items.b4** | **means** | **F1** | **Variable** |
| --- | --- | --- | --- | --- | --- | --- | --- |
| 5.800 | -0.468 | 1.102 | 2.033 | 2.636 | 0 | 1 | CPHQ2.0_3_moneydothings |
| 6.089 | -0.250 | 1.367 | 2.097 | 2.776 | 0 | 1 | CPHQ2.0_2_moneyhealthfood |

# 3. Person Score Distribution

| **Var1** | **Var2** | **Freq** |
| --- | --- | --- |
|  | F1 | Min. :-1.1274657 |
|  | F1 | 1st Qu.:-1.1274657 |
|  | F1 | Median : 0.3632029 |
|  | F1 | Mean : 0.0002322 |
|  | F1 | 3rd Qu.: 0.3632029 |
|  | F1 | Max. : 3.0272539 |

# 4. Item Loadings and Communalities

| **F1** | **h2** | **F1.1** | **Variable** |
| --- | --- | --- | --- |
| 0.960 | 0.921 | 1 | CPHQ2.0_3_moneydothings |
| 0.963 | 0.928 | 1 | CPHQ2.0_2_moneyhealthfood |

**Part 7:** IRT results for Health Literacy

# 1. Model Fit Indices

| **Model** | **AIC** | **BIC** | **HQ** | **SABIC** | **logLik** | **chi_square** | **p_value** |
| --- | --- | --- | --- | --- | --- | --- | --- |
| GPCM | 1,625.42 | 1,663.351 | 1,640.304 | 1,634.784 | -803.71 | 23.519 | 0.009 |

# 2. Item Parameters

| **items.a** | **items.b1** | **items.b2** | **items.b3** | **items.b4** | **means** | **F1** | **Variable** |
| --- | --- | --- | --- | --- | --- | --- | --- |
| 2.116 | -0.073 | 2.314 | 3.457 |  | 0 | 1 | CPHQ2.0_22_understandinfo |
| 2.044 | -1.036 | 1.586 | 2.747 | 3.199 | 0 | 1 | CPHQ2.0_21_findhelp |

# 3. Person Score Distribution

| **Var1** | **Var2** | **Freq** |
| --- | --- | --- |
|  | F1 | Min. :-1.2100421 |
|  | F1 | 1st Qu.:-0.3703341 |
|  | F1 | Median : 0.4371745 |
|  | F1 | Mean :-0.0001952 |
|  | F1 | 3rd Qu.: 0.4371745 |
|  | F1 | Max. : 2.7918098 |

# 4. Item Loadings and Communalities

| **F1** | **h2** | **F1.1** | **Variable** |
| --- | --- | --- | --- |
| 0.779 | 0.607 | 1 | CPHQ2.0_22_understandinfo |
| 0.768 | 0.591 | 1 | CPHQ2.0_21_findhelp |

**Part 8:** IRT results for Physical self-efficacy

# 5. Model Fit Indices

| **Model** | **AIC** | **BIC** | **HQ** | **SABIC** | **logLik** | **chi_square** | **p_value** |
| --- | --- | --- | --- | --- | --- | --- | --- |
| GPCM | 1,785.672 | 1,823.603 | 1,800.556 | 1,795.036 | -883.836 | 33.108 | 0 |

# 6. Item Parameters

| **items.a** | **items.b1** | **items.b2** | **items.b3** | **items.b4** | **means** | **F1** | **Variable** |
| --- | --- | --- | --- | --- | --- | --- | --- |
| 1.922 | -0.688 | 1.737 | 2.539 |  | 0 | 1 | CPHQ2.0_17_canmove |
| 4.709 | -0.849 | 0.992 | 2.005 | 2.668 | 0 | 1 | CPHQ2.0_20_canbeactive |

# 7. Person Score Distribution

| **Var1** | **Var2** | **Freq** |
| --- | --- | --- |
|  | F1 | Min. :-1.4105360 |
|  | F1 | 1st Qu.:-0.3551066 |
|  | F1 | Median : 0.1363774 |
|  | F1 | Mean :-0.0003127 |
|  | F1 | 3rd Qu.: 0.1363774 |
|  | F1 | Max. : 2.5852323 |

# 8. Item Loadings and Communalities

| **F1** | **h2** | **F1.1** | **Variable** |
| --- | --- | --- | --- |
| 0.749 | 0.560 | 1 | CPHQ2.0_17_canmove |
| 0.940 | 0.884 | 1 | CPHQ2.0_20_canbeactive |
